# Draft Genome Sequence of a Deep-Sea Bacterium, *Bacillus niacini* Strain JAM F8, Involved in the Degradation of Glycosaminoglycans

**DOI:** 10.1128/genomeA.00983-14

**Published:** 2014-10-02

**Authors:** Atsushi Kurata, Midori Nishimura, Noriaki Kishimoto, Tohru Kobayashi

**Affiliations:** a Department of Applied Biological Chemistry, Graduate School of Agriculture, Kindai University, Nara, Japan; b Research and Development Center for Marine Biosciences, Japan Agency for Marine-Earth Science and Technology, Yokosuka, Japan

## Abstract

Here, we report the draft genome sequence of *Bacillus niacini* JAM F8, which was newly isolated from deep-sea sediment at a depth of 2,759 m from the Izu-Ogasawara Trench. An array of genes related to degradation of glycosaminoglycans in this bacterium was identified by whole-genome analysis.

## GENOME ANNOUNCEMENT

Glycosaminoglycans are complex polysaccharides that are composed of disaccharide-repeating units, including one uronic acid and one amino sugar, and are present in the extracellular matrix in all animals (1, 2). The polysaccharides are categorized as sulfated glycans (chondroitin sulfate, dermatan sulfate, heparin sulfate, heparan sulfate, and keratan sulfate) or an unsulfated glycan (hyaluronic acid) (3, 4). A deep-sea bacterium, *Bacillus niacini* JAM F8 (JCM19735, 16S rRNA gene GenBank accession number AB889607), was isolated from deep-sea sediment from the Izu-Ogasawara Trench (30° 07.05N, 139° 58.42E) at a depth of 2,759 m. The strain JAM F8 utilizes chondroitin sulfate C as a carbon source and produces an enzyme that obviously degrades chondroitin sulfates A and C, chondroitin, and hyaluronic acid. The detailed biological data of the enzyme will be published elsewhere. Various glycosaminoglycan-degrading bacteria have been isolated to date, and various enzymes involved in the degradation of the polysaccharides were identified (5–7). Only three enzymes—heparinase (8), hyaluronidase (9), and unsaturated glucuronyl hydrolase (10)—have been identified in bacteria belonging to the genus *Bacillus*. In order to elucidate the enzymes involved in the degradation of glycosaminoglycans in *B. niacini* JAM F8, whole-genome sequence analysis was performed.

The genome of *B. niacini* JAM F8 was sequenced using the Illumina HiSeq (Hokkaido System Science Co., Ltd., Sapporo, Japan). Paired-end libraries of 100-bp fragments were prepared from the genomic DNA, and the genome sequencing generated 14,496,952 raw reads covering a total of 1,464 Mbp (Phred >Q30 = 96.76%). Quality-trimmed DNA reads conferring an average of 200-fold coverage were assembled *de novo* by Velvet 1/2/08. As a result, the draft genome sequence comprised 170 contigs totaling 6,372,252 bp with an average length of 37,484 bp (largest 637,777 bp and smallest 169 bp). An *N*_50_ contig length of 212,448 bp, and an *N*_90_ contig length of 55,357 bp were obtained. The total G+C content was 37.8%. All assembly data were deposited in the DDBJ/EMBL/GenBank/nucleotide sequence database.

The gene prediction and functional annotation were carried out by the Microbial Genome Annotation Pipeline (MiGAP, http://www.migap.org) (11), which utilizes MetaGeneAnnotator (12), RNAmmer (13), tRNAscan-SE (14), and NCBI BLAST (15). It predicted a total of 6,134 protein-encoding genes, 13 rRNA genes, and 129 tRNA genes.

We identified four genes encoding glycosaminoglycan-degrading enzymes by RAST annotation (http://blog.theseed.org/servers) (16), namely, a chondroitinase gene, a hyaluronidase gene, a hyaluronoglucosaminidase gene, and a heparinase gene. Furthermore, each gene comprises the gene cluster responsible for degradation of chondroitin sulfate (5 genes), hyaluronic acid (11 genes), or heparin (7 genes) to produce their corresponding monosaccharides. As a result, many of the 23 genes associated with utilization of glycosaminoglycans were found in the strain JAM F8 genome. We plan to detect other genes involved in the degradation of glycosaminoglycans in the genome of *B. niacini* JAM F8.

### Nucleotide sequence accession numbers.

The draft genome sequence of *B. niacini* JAM F8 is available at the DDBJ/EMBL/GenBank under the accession numbers BAWM01000001 through BAWM01000170.
